# The effect of campus bullying on negative emotions among middle school students: a moderated mediation model

**DOI:** 10.3389/fpubh.2026.1811136

**Published:** 2026-05-05

**Authors:** Zhoutong Dai, Yifan Hu, Cong Wang, Claire Mei, Yuting Wang, Jia Tao, Yaning Yang, Yan Liu, Zhiyuan Cheng, Dan Su

**Affiliations:** 1 School of Nursing, Anhui Medical University, Hefei, China; 2 Affiliated Psychological Hospital of Anhui Medical University, Anhui Medical University, Hefei Fourth People's Hospital, Hefei, China; 3 Tongji Medical College, Huazhong University of Science and Technology, Wuhan, China; 4 The Middle School Attached to the University of Science and Technology of China, Hefei, China; 5 Feixi County Third Middle School, Anhui Province, Hefei, China

**Keywords:** anxiety, core self-evaluation, depression, interpersonal relationship, middle school student, negative emotions, school bullying, stress

## Abstract

**Background:**

This study aimed to explore the mechanism by which school bullying among middle school students influences negative emotions and to test a moderated mediation model.

**Methods:**

An anonymous cross-sectional survey employed a convenience sampling method among secondary school students in Anhui Province, eastern China, conducted from October to December 2023. Data were collected using a sociodemographic information questionnaire, the Core Self-Evaluation Scale (CSES), the Depression-Anxiety-Stress Scale-21 (DASS-21), and the Secondary School Students' Interpersonal Relationships Questionnaire. Statistical analysis was performed using AMOS 24.0 and SPSS 25.0.

**Results:**

A total of 1,340 middle school students participated in this study. According to a simple mediation analysis based on Model 4, core self-evaluation partially mediated the relationships between school bullying and depression, anxiety, and stress, with indirect effect sizes of 0.32, 0.31, and 0.31, respectively, accounting for 37.67%, 34.07%, and 36.47% of the total effect. A moderated mediation analysis based on Model 14 further indicated that interpersonal relationships significantly moderated the relationship between core self-evaluation and depression, anxiety, and stress (interaction terms β = 0.11–0.22, *p* < 0.001), with the strongest moderating effect observed for depression. After distinguishing the various dimensions of interpersonal relationships, it was found that peer, teacher-student, parent-child, and self relationships all significantly moderated the relationship between core self-evaluation and depression, anxiety, and stress (interaction terms β = 0.06–0.22, *p* < 0.001), with the moderating effects of self and parent-child relationships being the most pronounced.

**Conclusion:**

This study found that core self-evaluation partially mediated the relationship between school bullying and depression, anxiety, and stress; interpersonal relationships significantly moderated this mediating pathway. These findings provide empirical evidence for understanding the mechanisms through which school bullying affects the negative emotions of middle school students and for implementing targeted interventions.

## 1 Introduction

The middle school years are a critical period for individual psychological development and mental health protection (1). In recent years, the incidence of school bullying has been high; as a school-related issue prevalent worldwide, it has become a significant challenge facing the public health and education sectors (2–5).

School bullying refers to situations where, in a context of unequal power dynamics, an individual intentionally and repeatedly subjects a peer with less power to physical, verbal, or psychological attacks with the intent of causing the victim fear, anxiety, or harm (6). Negative emotions refer to the negative emotional responses generated when an individual encounters negative stimuli or stress, including depression, anxiety, and stress (7). Depression is characterized by low self-esteem, a loss of motivation, and pessimistic expectations regarding life goals; anxiety is centered on fear and accompanied by physical and situational tension; stress refers to a state of persistent tension, irritability, and difficulty in calming down (8). Research indicated that school bullying is significantly positively correlated with depression, anxiety, and stress among middle school students and can predict the occurrence of these emotional issues (9, 10). Persistent or severe depression, anxiety, and stress not only impair mental health but may also increase the risk of suicidal ideation among middle school students (11, 12). Therefore, exploring the mechanisms by which school bullying leads to depression, anxiety, and stress in middle school students holds significant practical implications for mental health interventions.

In addition to direct relationships, it is also necessary to understand how variables are interconnected through indirect effects. Core self-evaluation is an individual's fundamental assessment of their intrinsic value and abilities (13) and serves as an internal resource for the mental health of middle school students. Judge et al. (14) pointed out that core self-evaluation exhibits characteristics of a relatively stable cognitive schema when individuals process external information. Specifically, individuals with high core self-evaluation exhibit a stronger sense of control over their lives and are more confident about the future (15); conversely, those with low core self-evaluation are more likely to focus on and internalize negative feedback, leading to negative cognitions and, consequently, depression, anxiety, and stress (16). Therefore, core self-evaluation may serve as a mediating factor in the relationship between school bullying and depression, anxiety, and stress.

Interpersonal relationships are direct psychological bonds formed through interaction and engagement between people, including peer, teacher-student, parent-child, and self-relationships (17). Peer relationships refer to equal interactions and emotional bonds among peers; teacher-student relationships involve interactions between students and teachers regarding academics and behavioral norms; parent-child relationships refer to emotional bonds and support between parents and children; and self-relationships refer to an individual's fundamental evaluation of their own worth and abilities (18). According to social support theory (19), interpersonal relationships and social networks can promote mental health, and interpersonal relationships serve as external resources for the mental health of middle school students (20). Therefore, interpersonal relationships may play a moderating role between core self-evaluation and depression, anxiety, and stress.

**Figure 1:**
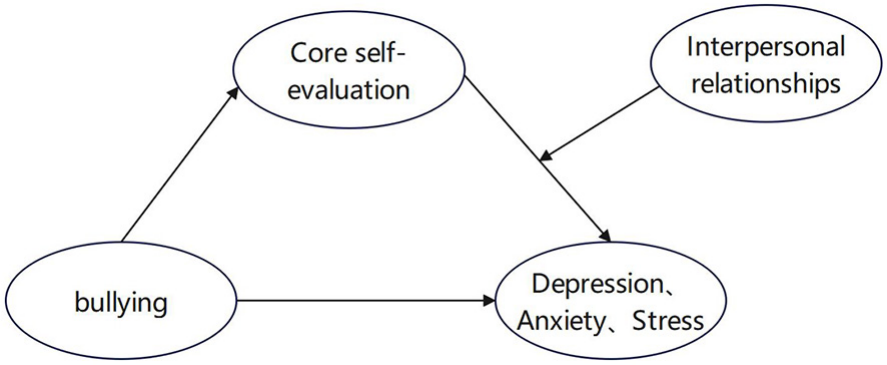
The hypothesized model.

Based on the above analysis, this study aimed to construct a moderated mediation model to systematically examine the effects of school bullying on depression, anxiety, and stress among middle school students. It tested the mediating role of core self-evaluation and the moderating effects of four relationship dimensions—peer, teacher-student, parent-child, and self—with the goal of providing empirical support for the early detection and targeted intervention of depression, anxiety, and stress in middle school students. This study proposed the following hypotheses, as shown in Figure 1:

H1: School bullying positively predicts depression, anxiety, and stress among middle school students.
H2: Core self-evaluation mediates the relationship between school bullying and depression, anxiety, and stress.
H3: Interpersonal relationships moderate the relationship between core self-evaluation and depression, anxiety, and stress.

## 2 Methods

### 2.1 Sample

The research participants were selected from 6 middle schools in Anhui Province, China, including five middle schools in Hefei City and one middle school in Huangshan City. A total of 1,340 Anhui secondary school students were selected between November 2023 and December 2023 using convenience sampling. Among the study participants, 45.8% were female (*N* = 614) and 54.2% were male (*N* = 726). The average age was 15.06 years (SD = 1.36), with ages ranging from 12 to 20 years.

### 2.2 Programs

In accordance with the Declaration of Helsinki, all participants and their legal guardians were fully aware of the purpose and content of the study and provided written consent prior to their participation. The study was approved by the Ethics Review Committee of the Anhui Medical University (Ethics Approval Number: 82240186). This survey was conducted voluntarily and anonymously, and participants could voluntarily withdraw at any stage. The questionnaire was administered in paper form, distributed during self-study or rest periods, and completed on the spot. It took approximately 15 min. Participants were required to complete a questionnaire to assess campus bullying, core self-evaluation, depression, anxiety, and stress, and interpersonal relationships. After the questionnaires were collected, the questionnaires were checked one by one. If there were consistent answer choices, omitted questions more than 1/3 of the total number of questions, or regularity in the choices, the questionnaires were regarded as invalid and were excluded. To ensure the accuracy of data entry, two people were responsible for data entry. This study ultimately collected 1,340 valid questionnaires, with an effective response rate of 94.4%.

### 2.3 Instruments

#### 2.3.1 General information

A general information questionnaire designed by the researchers, which included grade level, academic performance, and whether the respondent had experienced school bullying.

#### 2.3.2 School bullying

This was a single-choice question from a questionnaire designed by the researcher. The question asked whether the respondent had ever been bullied at school, with a binary response scale: 1 (Yes) and 2 (No).

#### 2.3.3 Core Self-Evaluations Scale(CSES)

Developed by Judge in 2003 (21) and revised by Du Jianzheng in 2006 (18), this scale measured an individual's core self-evaluation of their own abilities and worth. It comprised four dimensions—self-esteem, general self-efficacy, emotional stability, and internal locus of control—and featured a unidimensional structure with a total of 10 items. It employed a 5-point Likert scale ranging from 1 (strongly disagree) to 5 (strongly agree). Items 2, 3, 5, 7, 8, and 10 were reverse-scored. The cumulative total of all item scores constituted the final score, which ranged from 10 to 50 points; a higher score indicated a higher level of the respondent's core self-evaluation. The Cronbach's α for this questionnaire was 0.83 (18). In this study, the Cronbach's α for this questionnaire was 0.883.

#### 2.3.4 Depression Anxiety and Stress Scale-21(DASS-21)

Developed by Lovibond et al. in 1993 (8), this scale was used to assess the severity of three negative emotional states—depression, anxiety, and stress—in individuals over the past week. It consisted of three dimensions and 21 items, divided into depression (7 items: 3, 5, 10, 13, 16, 17, 21), anxiety (7 items: 2, 4, 7, 9, 15, 19, 20), and stress (7 items: 1, 6, 8, 11, 12, 14, 18). A 4-point Likert scale was used, ranging from 0 (strongly disagree) to 3 (strongly agree). The total score for each dimension multiplied by 2 yielded the score for depressive symptoms, anxiety symptoms, and stress symptoms, respectively. Higher scores indicated more severe symptoms; scores of ≥10, ≥8, and ≥15 indicated the presence of depressive symptoms, anxiety symptoms, and stress symptoms, respectively. The Cronbach's α for the depression, anxiety, and stress dimensions of the scale were 0.91, 0.84, and 0.90, respectively (8). In this study, the Cronbach's α for the depression, anxiety, and stress dimensions of the scale were 0.882, 0.855, and 0.866, respectively.

#### 2.3.5 Interpersonal relationship questionnaire for middle school students

Developed by Wu Chao (22), this scale was used to assess the quality of relationships among middle school students across four dimensions: peer, parent-child, teacher-student, and self. It consisted of four dimensions and 20 items, divided into peer relationships (5 items: 1–5), teacher-student relationships (7 items: 6–12), parent-child relationships (4 items: 13–16), and self-relationships (4 items: 17–20). It employed a 5-point Likert scale ranging from 0 (strongly disagree) to 4 (strongly agree). The cumulative total of all item scores constituted the final score, with a total score range of 0–80 points; a higher score indicated better interpersonal relationships for the respondent. The questionnaire's raw Cronbach's α was 0.892, indicating good reliability (22); in this study, the Cronbach's α for this questionnaire was 0.920.

### 2.4 Statistical analysis

Data analysis in this study was conducted using AMOS 24.0 and SPSS 25.0. AMOS 24.0 was used for confirmatory factor analysis to assess the structural validity of the measurement model. SPSS was used to perform Harman's single-factor test, descriptive statistics, and correlation analysis. Additionally, PROCESS 4.0 (Model 4) was used to examine the mediating role of core self-evaluation in the relationship between school bullying and depression, anxiety, and stress. Finally, PROCESS 4.0 (Model 14) was employed to analyze the moderated mediation model. This study utilized the bootstrap method to estimate confidence intervals (CI) based on 5,000 bootstrap samples.

The mediation analysis procedure was as follows: (1) Descriptive statistics and correlation analysis were conducted on school bullying, core self-evaluation, depression, anxiety, stress, interpersonal relationships and their dimensions; (2) Tested the mediating effect of core self-evaluation on the relationship between school bullying and depression, anxiety, and stress; (3) Tested the moderating effect of interpersonal relationships on the mediating path between core self-evaluation and depression, anxiety, and stress; (4) Further examined the differential moderating effects of the four dimensions of interpersonal relationships. All analyses were conducted using SPSS 25.0.

## 3 Results

### 3.1 Confirmatory factor analysis

This study employed confirmatory factor analysis to assess the structural validity of the measurement model. Although the large sample size resulted in a relatively high χ^2^/df ratio, the overall model demonstrated good fit (23); details were presented in Table 1.

**Table 1:** Confirmatory factor analysis.

Fit index	DASS-21	IRS	CSES	Reference value
*x*^2^/df	8.323	6.258	9.501	< 3
RMSEA	0.074	0.063	0.080	< 0.08
GFI	0.917	0.936	0.951	>0.90
TLI	0.906	0.926	0.931	>0.90
SRMR	0.040	0.048	0.045	< 0.08

### 3.2 Common method bias test

Harman's one-factor test showed that the eigenvalues of all seven principal factors were greater than 1, and the first principal factor accounted for 33.96% of the variance (less than 40%). Therefore, potential concerns regarding common-method bias in this study were considered negligible.

### 3.3 Preliminary analyses

The results of the descriptive and correlational analyses were presented in Table 2. School bullying was significantly negatively correlated with core self-evaluation, interpersonal relationships and their dimensions; it was significantly positively correlated with depression, anxiety, and stress. Core self-evaluation was significantly negatively correlated with depression, anxiety, and stress, and was significantly positively correlated with interpersonal relationships and their dimensions. Interpersonal relationships and their dimensions were all significantly negatively correlated with depression, anxiety, and stress. H1 was supported.

**Table 2:** Descriptive and correlational analysis.

Variable	Statistic		Correlations									
	*M*	*SD*	1	2	3	4	5	6	7	8	9	10
1.School bullying	0.07	0.26	–	–	–	–	–	–	–	–	–	–
2.Core self-evaluation	32.99	7.55	−0.15^**^	–	–	–	–	–	–	–	–	–
3.Depression	6.76	8.66	0.23^**^	−0.65^**^	–	–	–	–	–	–	–	–
4.Anxiety	8.43	8.79	0.24^**^	−0.63^**^	0.83^**^	–	–	–	–	–	–	–
5.Stress	9.46	9.26	0.23^**^	−0.63^**^	0.82^**^	0.86^**^	–	–	–	–	–	–
6.Interpersonal relationships	45.02	15.18	−0.14^**^	0.62^**^	−0.52^**^	−0.45^**^	−0.46^**^	–	–	–	–	–
7.Companion	12.83	4.23	−0.08^*^	0.42^**^	−0.36^**^	−0.33^**^	−0.34^**^	0.73^**^	–	–	–	–
8.Teachers and students	13.83	6.59	−0.10^**^	0.46^**^	−0.36^**^	−0.32^**^	−0.32^**^	0.86^**^	0.48^**^	–	–	–
9.Parents and child	9.09	4.29	−0.15^**^	0.48^**^	−0.44^**^	−0.38^**^	−0.38^**^	0.79^**^	0.44^**^	0.56^**^	–	–
10.self	9.28	3.84	−0.13^**^	0.65^**^	−0.54^**^	−0.47^**^	−0.46^**^	0.78^**^	0.48^**^	0.55^**^	0.56^**^	–

^*^p < 0.05,

^**^p < 0.01,

^***^p < 0.001.

### 3.4 Mediation analyses

The path coefficients for the mediation model were presented in Table 3, and the results of the effect decomposition were shown in Table 4. The results of the mediation analysis indicated that, after controlling for gender, grade level, and academic performance, core self-evaluation partially mediated the relationship between school bullying and depression, anxiety, and stress among middle school students. Specifically, school bullying not only directly and positively predicted depression, anxiety, and stress (depression: β = 0.53, 95% CI [0.37, 0.68]; anxiety: β = 0.60, 95% CI [0.44, 0.76]; stress: β = 0.53, 95% CI [0.38, 0.69]), but also exerted a significant indirect effect via core self evaluation (indirect effect on depression = 0.32, 95% CI [0.20, 0.45]; indirect effect on anxiety = 0.31, 95% CI [0.19, 0.43]; stress indirect effect = 0.31, 95% CI [0.19, 0.43]). The proportion of the total effect accounted for by the mediating effects was 37.65% for depression, 34.07% for anxiety, and 36.47% for stress, with the depression model exhibiting the highest proportion of the mediating effect. H2 was supported.

**Table 3:** Results of the mediation models.

Outcomes	Predictors	β	SE	*T*	95% CI
Depression	Gender	−0.07	0.04	−1.58	[−0.15, 0.02]
	Grade	0.09^***^	0.02	5.10	[0.05, 0.12]
	Academic record	−0.03	0.02	−1.49	[−0.08, 0.01]
	School bullying	0.53^***^	0.08	6.68	[0.37, 0.68]
	Core self-evaluation	−0.62^***^	0.02	−27.37	[−0.67, −0.58]
	*R^2^*	0.46^***^			
	*F*	224.17			
Anxiety	Gender	0.03	0.04	0.79	[−0.05, 0.12]
	Grade	0.10^***^	0.02	5.89	[0.07, 0.13]
	Academic record	−0.08^***^	0.02	−3.70	[−0.13, −0.04]
	School bullying	0.60^***^	0.08	7.48	[0.44, 0.76]
	Core self-evaluation	−0.60^***^	0.02	−25.94	[−0.64, −0.55]
	*R^2^*	0.44^***^			
	*F*	208.91			
Stress	Gender	0.00	0.04	−0.07	[−0.09, 0.08]
	Grade	0.12^***^	0.02	6.78	[0.08, 0.15]
	Academic record	−0.08^***^	0.02	−3.77	[−0.13, −0.04]
	School bullying	0.53^***^	0.08	6.65	[0.38, 0.69]
	Core self-evaluation	−0.60^***^	0.02	−25.94	[−0.64, −0.55]
	*R^2^*	0.44^***^			
	*F*	208.04			

^*^p < 0.05,

^**^p < 0.01,

^***^p < 0.001.

**Table 4:** Table of mediator analysis results.

Outcomes	Effect type	β	SE	BootSE	*t*	95% CI	%
Depression	*c*	0.85^***^	0.10	—	8.70	[0.66, 1.04]	100%
	*c'*	0.53^***^	0.08	—	6.68	[0.37, 0.68]	62.35%
	*ab*	0.32^***^	—	0.06	—	[0.20, 0.45]	37.65%
Anxiety	*c*	0.91^***^	0.10	—	9.35	[0.72, 1.10]	100%
	*c'*	0.60^***^	0.08	—	7.48	[0.44, 0.76]	65.93%
	*ab*	0.31^***^	—	0.06	—	[0.19, 0.43]	34.07%
Stress	*c*	0.85^***^	0.01	—	8.67	[0.65, 1.04]	100%
	*c'*	0.53^***^	0.08	—	6.65	[0.38, 0.69]	62.35%
	*ab*	0.31^***^	—	0.06	—	[0.19, 0.43]	36.47%

Independent variable: school bullying; Mediator: core self-evaluation.

c = total effect; c' = direct effect; ab = indirect effect.

^*^p < 0.05,

^**^p < 0.01,

^***^p < 0.001.

### 3.5 Moderated mediation analyses

The results of the moderated mediation analysis are shown in Table 5. After controlling for gender, grade, and academic performance, school bullying had a significant negative predictive effect on core self-evaluation (β = −0.52, *p* < 0.001). The interaction term between core self-evaluation and interpersonal relationships had a significant positive predictive effect on depression, anxiety, and stress (β = 0.11–0.22, *p* < 0.001), indicating that interpersonal relationships significantly moderated the association between core self-evaluation and depression, anxiety, and stress.

**Table 5:** Results of the moderated mediation models.

Outcomes	Predictors	β	SE	*T*	95% CI
Core self-evaluation	Gender	−0.38^***^	0.05	−7.57	[−0.47, −0.28]
	Grade	−0.17^***^	0.02	−8.59	[−0.21, −0.13]
	Academic record	−0.30^***^	0.03	−12.14	[−0.35, −0.25]
	School bullying	−0.52^***^	0.09	−5.50	[−0.70, −0.33]
	*R^2^*	0.21^***^			
	*F*	87.54			
Depression	Gender	−0.06	0.04	−1.49	[−0.13, 0.02]
	Grade	0.09^***^	0.02	5.75	[0.06, 0.12]
	Academic record	−0.06^*^	0.02	−2.72	[−0.10, −0.02]
	School bullying	0.44^***^	0.07	5.97	[0.29, 0.58]
	Core self-evaluation	−0.51^***^	0.03	−20.34	[−0.56, −0.46]
	Interpersonal relationships	−0.19^***^	0.02	−7.84	[−0.24, −0.14]
	Int	0.22^***^	0.02	13.57	[0.19, 0.25]
	*R^2^*	0.54^***^			
	*F*	220.86			
	*W* −1 *SD*	0.38	0.07	–	[0.23, 0.53]
	*W*	0.27	0.06	–	[0.17, 0.38]
	*W* +1 *SD*	0.15	0.03	–	[0.09, 0.22]
Anxiety	Gender	0.04	0.04	0.93	[−0.04, 0.12]
	Grade	0.10^***^	0.02	6.16	[0.07, 0.14]
	Academic record	−0.10^***^	0.02	−4.36	[−0.14, −0.05]
	School bullying	0.55^***^	0.08	6.96	[0.39, 0.70]
	Core self-evaluation	−0.53^***^	0.03	−19.83	[−0.59, −0.48]
	Interpersonal relationships	−0.11^***^	0.03	−4.22	[−0.16, −0.06]
	Int	0.13^***^	0.02	7.49	[0.10, 0.16]
	*R^2^*	0.47^***^			
	*F*	166.80			
	*W*−1 *SD*	0.34	0.07	–	[0.21, 0.48]
	*W*	0.28	0.06	–	[0.17, 0.40]
	*W* + 1 *SD*	0.21	0.05	–	[0.13, 0.31]
Stress	Gender	0.00	0.04	0.08	[−0.08, 0.09]
	Grade	0.12^***^	0.02	6.96	[0.08, 0.15]
	Academic record	−0.10^***^	0.02	−4.44	[−0.14, −0.05]
	School bullying	0.49^***^	0.08	6.14	[0.33, 0.64]
	Core self-evaluation	−0.53^***^	0.03	−19.60	[−0.58, −0.48]
	Interpersonal relationships	−0.12^***^	0.03	−4.45	[−0.17, −0.06]
	Int	0.11^***^	0.02	6.40	[0.08, 0.15]
	*R^2^*	0.46^***^			
	*F*	162.89			
	*W*−1 *SD*	0.33	0.07	–	[0.21, 0.47]
	*W*	0.28	0.06	–	[0.17, 0.39]
	*W* + 1 *SD*	0.22	0.05	–	[0.13, 0.31]

^*^p < 0.05,

^**^p < 0.01,

^***^p < 0.001.

The analysis of conditional indirect effects further confirmed the moderating effect: when the level of interpersonal relationships was low (−1 *SD*), the indirect effects of school bullying on depression, anxiety, and stress through core self-evaluation were 0.38 (95% CI [0.23, 0.53]), 0.34 (95% CI [0.21, 0.48]), and 0.33 (95% CI [0.21, 0.47]), respectively; when the level of interpersonal relationships was high (+1 *SD*), the indirect effects decreased to 0.15 (95% CI [0.09, 0.22]), 0.21 (95% CI [0.13, 0.31]), and 0.22 (95% CI [0.13, 0.31]), respectively.

**Figure 2:**
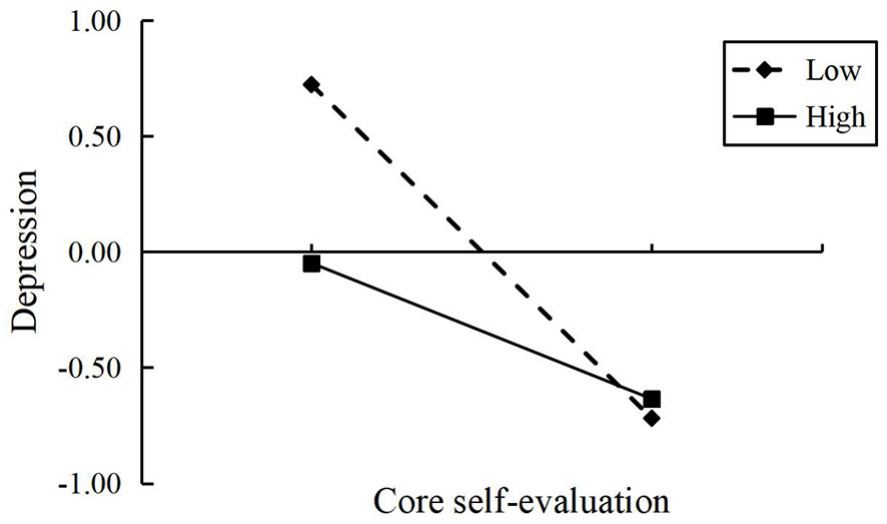
The moderating effect of interpersonal relationships on the association between core self-evaluation and depression.

**Figure 3:**
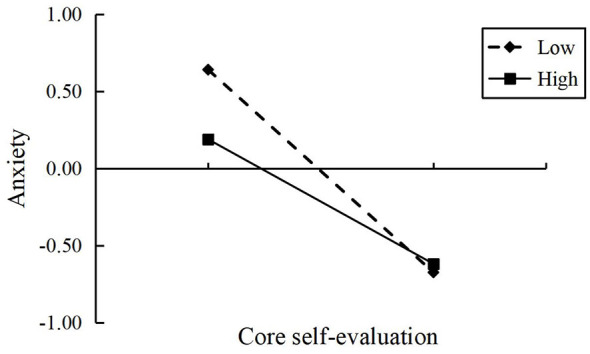
The moderating effect of interpersonal relationships on the association between core self-evaluation and anxiety.

**Figure 4:**
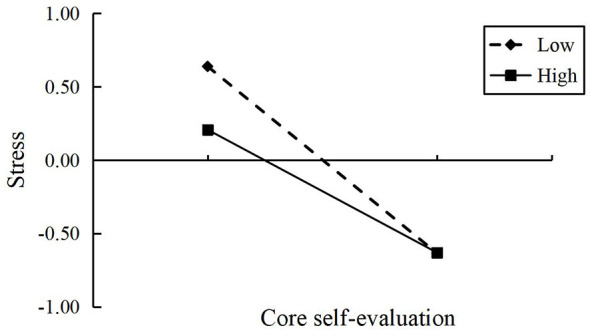
The moderating effect of interpersonal relationships on the association between core self-evaluation and stress.

Simple slope analysis further revealed the direction of the moderation effect: as shown in Figures 2–4, when the level of interpersonal relationships was high (+1 *SD*), the negative predictive effect of core self-evaluation on depression, anxiety, and stress was weakened compared to when the level of interpersonal relationships was low (−1 *SD*).

In summary, interpersonal relationships significantly moderated the indirect pathways through which school bullying affected depression, anxiety, and stress through core self-evaluation. A high level of interpersonal relationships reduced this mediating effect, while a low level of interpersonal relationships enhanced it, with the most prominent moderating effect on depression. H3 was supported.

### 3.6 Mediational analysis of various dimensions of interpersonal relationships

As shown in Table 6, the interaction terms between core self-evaluation and various dimensions of interpersonal relationships had significant positive predictive effects on depression, anxiety, and stress (β = 0.06–0.22, *p* < 0.001), indicating that all dimensions of interpersonal relationships significantly moderated the association between core self-evaluation and depression, anxiety, and stress.

**Table 6:** Mediational analysis of various dimensions of interpersonal relationships.

Outcomes	Predictors	Companion	Teachers and students	Parents and child	self
Depression	Gender	−0.04	−0.07	−0.07	−0.08
	Grade	0.09^***^	0.09^***^	0.09^***^	0.09^***^
	Academic record	−0.04	−0.04	−0.04	−0.06^**^
	School bullying	0.51^***^	0.50^***^	0.41^***^	0.44^***^
	Core self–evaluation	−0.57^***^	−0.60^***^	–.057^***^	−0.50^***^
	Interpersonal relationship dimension	−0.11^***^	−0.08^***^	−0.13^***^	−0.16^***^
	Int	0.16^***^	0.15^***^	0.20^***^	0.22^***^
	*R* ^2^	0.50^***^	0.49^***^	0.52^***^	0.54^***^
	*F*	189.51	181.51	205.73	223.32
	*W* – 1 *SD*	0.37	0.39	0.40	0.39
	*W*	0.29	0.31	0.30	0.27
	*W* + 1 *SD*	0.21	0.22	0.18	0.13
Anxiety	Gender	0.05	0.03	0.03	0.03
	Grade	0.11^***^	0.10^***^	0.10^***^	0.11^***^
	Academic record	−0.09^***^	−0.09^***^	−0.08^***^	−0.10^***^
	School bullying	0.59^***^	0.59^***^	0.54^***^	0.54^***^
	Core self-evaluation	−0.56^***^	−0.59^***^	−0.57^***^	−0.53^***^
	Interpersonal relationship dimension	−0.08^***^	−0.03	−0.07^***^	−0.09^***^
	Int	0.11^***^	0.08^***^	0.11^***^	0.15^***^
	*R^2^*	0.46^***^	0.45^***^	0.46^***^	0.47^***^
	*F*	162.36	154.12	160.47	170.12
	*W* −1 *SD*	0.35	0.35	0.35	0.36
	*W*	0.29	0.30	0.30	0.28
	*W* +1 *SD*	0.23	0.26	0.23	0.19
Stress	Gender	0.02	−0.01	0.00	−0.01
	Grade	0.12^***^	0.12^***^	0.12^***^	0.12^***^
	Academic record	−0.09^***^	−0.09^***^	−0.09^***^	−0.10^***^
	School bullying	0.52^***^	0.52^***^	0.47^***^	0.48^***^
	Core self-evaluation	−0.56^***^	−0.58^***^	−0.57^***^	−0.54^***^
	Interpersonal relationship dimension	−0.09^***^	−0.04	−0.08^***^	−0.08^***^
	Int	0.09^***^	0.06^***^	0.10^***^	0.14^***^
	*R* ^2^	0.45^***^	0.44^***^	0.46^***^	0.47^***^
	*F*	158.12	151.67	159.74	166.22
	*W* – 1 *SD*	0.33	0.34	0.35	0.36
	*W*	0.29	0.30	0.30	0.28
	*W* +1 *SD*	0.25	0.27	0.23	0.20

^*^p < 0.05,

^**^p < 0.01,

^***^p < 0.001.

The analysis of conditional indirect effects further confirmed the moderating effect: when the levels of peer, teacher-student, parent-child, and self-relationship were low (−1 *SD*), the indirect effects on depression were 0.37, 0.39, 0.40, and 0.39, respectively; the indirect effects on anxiety were 0.35, 0.35, 0.35, and 0.36, respectively; the indirect effects on stress were 0.33, 0.34, 0.35, and 0.36, respectively. When the levels of each dimension were high (+1 *SD*), the indirect effects on depression decreased to 0.21, 0.22, 0.18, and 0.13, respectively; the indirect effects on anxiety decreased to 0.23, 0.26, 0.23, and 0.19, respectively; the indirect effects on stress decreased to 0.25, 0.27, 0.23, and 0.20, respectively.

Overall, all dimensions of interpersonal relationships significantly moderated the indirect pathways through which school bullying affected depression, anxiety, and stress through core self-evaluation: higher levels of interpersonal relationships reduced this mediating effect, while lower levels increased it. Among them, the moderating effect of self-relationship was the strongest, followed by parent-child relationship.

## 4 Discussion

First, this study found that middle school students' anxiety scores were relatively high (8.43 ± 8.79 > 8 points), suggesting that anxiety is prevalent among middle school students in the Chinese educational context, consistent with the findings of Wang et al. (24). This may be related to factors such as academic burden and further education pressure (25). In this study, the overall level of interpersonal relationships among middle school students was found to be slightly above average. Among these, peer, parent-child, and self relationships were relatively good, while teacher-student relationships were relatively weak (13.83 ± 6.59 < 14), consistent with previous research (26). This may be related to China's exam-oriented education system, where teacher-student relationships often focus on students' academic performance (27). Furthermore, the prevalence of school bullying in this study was approximately 7%, which was lower than that reported in domestic studies (28, 29). This discrepancy may stem from: (1) Differences in measurement tools and definitions of bullying; (2) Variations in sample regions and school types; (3) Self-reporting methods may lead to underreporting of bullying.

Second, this study found that school bullying positively predicted depression, anxiety, and stress among middle school students; that is, students who experienced more bullying were more likely to exhibit negative emotional reactions such as anxiety, depression, and stress. This is consistent with the findings of previous studies (28, 30), and H1 was supported. Among them, Zhao et al., based on a large-scale survey of 95,545 Chinese adolescents, confirmed together with the present study that school bullying is an important risk factor that impairs the mental health of middle school students. The study clarified the universality of the psychological harm caused by bullying from an epidemiological perspective. This study further revealed the internal mediation and regulation mechanisms of bullying affecting depression, anxiety, and stress in middle school students. The differences between the two studies primarily stem from the following factors: first, sample characteristics: the former study surveyed students across all educational levels from elementary school through high school, whereas the present study focused primarily on middle and high school students. Second, regional differences: the two studies were conducted in China's southwestern and eastern regions, respectively, where there are regional variations in school mental health service systems and social support resources available to students. Third, differences in measurement methods: the former study employed a multidimensional scale, while the present study used a dichotomous variable, among other distinctions. During the secondary school years, students' self-awareness develops rapidly, they are highly sensitive to external evaluations, and their emotional regulation abilities are still in the process of development and refinement (31). Repeated exposure to bullying in school settings can lead to persistent psychological stress, which in turn exacerbates depression, anxiety, and stress. Therefore, in addition to addressing bullying behaviors, schools should place greater emphasis on its psychological consequences.

Third, the results of this study also confirmed that core self-evaluation mediated the relationship between school bullying and depression, anxiety, and stress; school bullying exacerbated depression, anxiety, and stress by lowering core self-evaluation, and H2 was supported. This finding is consistent with Cognitive Appraisal Theory (32), which posits that stressful events influence emotional responses by altering an individual's cognitive appraisal. As a stable structure of self-perception, core self-evaluation played a key mediating role between experiences of bullying and depression, anxiety, and stress, revealing the cognitive mechanisms through which school bullying affected depression, anxiety, and stress. Notably, the mediating effects of core self-evaluation were in the order of depression, stress, and anxiety, with depression accounting for the largest proportion of the mediating effect. According to the Cognitive Theory of Depression (33), the essence of depression lies in an individual's negative perceptions of the self, the world, and the future; therefore, as a form of self-perception, core self-evaluation played the strongest mediating role. Therefore, In addition to behavioral interventions, school bullying interventions can also reduce depression, anxiety, and stress by enhancing students' core self-evaluation.

Fourth, the results of this study found that interpersonal relationships moderated the relationship between core self-evaluation and depression, anxiety, and stress. When interpersonal relationships were positive, the influence of core self-evaluation on depression, anxiety, and stress was significantly reduced, supported H3. According to Conservation of Resources Theory (34), when an individual possesses multiple psychological resources simultaneously, there may be a substitution or compensatory relationship among these resources. Good interpersonal relationships, as an important external resource, can effectively reduce depression, anxiety, and stress on their own; in this situation, the individual's dependence on internal resources for core self-evaluation decreases, thereby reducing the effectiveness of core self-evaluation. On the contrary, when interpersonal relationships are poor, individuals lack sufficient external support and must highly rely on core self-evaluation to reduce depression, anxiety, and stress, thus enhancing the effect of core self-evaluation. Therefore, improving interpersonal relationships can compensate for a lack of internal resources, providing a theoretical basis for personalized interventions.

The moderating effects of interpersonal relationships on depression, anxiety, and stress varied, with depression having the strongest moderating effect, followed by stress and anxiety. This difference may stem from variations in the extent to which depression, anxiety, and stress influence reliance on interpersonal relationships (26). The results of this study indicated that, compared with anxiety and stress, depression was more significantly correlated with peer, teacher-student, parent-child, and self-relationships. Furthermore, core symptoms of depression include social avoidance and interpersonal alienation (35), making individuals more susceptible to the influence of interpersonal relationship quality and more dependent on them. In contrast, anxiety stems more from uncertainty about the future (36), whereas stress primarily arises from physiological, behavioral, and cognitive responses to external stressors (37) and is relatively less dependent on interpersonal relationships. Therefore, given the differences in the moderating effects of interpersonal relationships on depression, anxiety, and stress, schools should implement personalized mental health interventions for middle school students.

This study further distinguished among different dimensions of interpersonal relationships. The results indicated that peer, teacher-student, parent-child, and self-relationships all significantly moderated the relationship between core self-evaluation and depression, anxiety, and stress. However, there were significant differences in the strength of moderation across dimensions: the moderating effect of self-relationships was the most pronounced, followed by parent-child and peer relationships, whereas the moderating effect of teacher-student relationships was the weakest. According to Ecological Systems Theory (38), self-relationships represent the individual's internal characteristics as the subject. Parent-child, peer, and teacher-student relationships all belong to the microsystem. From the perspective of Developmental Psychology, parent-child relationships rooted in Attachment Theory are the earliest to form and carry the strongest emotional bonds (39). Peer relationships provide support through social acceptance and a sense of belonging (40), whereas teacher-student relationships focus more on academic performance and social adjustment (41). Therefore, the closer the psychological connection is to middle school students, the stronger the moderating effect is. Accordingly, personalized mental health interventions should be implemented for middle school students based on the differential moderating roles of self, parent-child, peer, and teacher-student relationships.

## 5 Conclusion

This study explores the underlying mechanisms linking school bullying among middle school students to negative emotions within the Chinese educational context. First, anxiety is particularly prevalent among middle school students, and teacher-student relationships are relatively weak. Second, the study finds that core self-evaluation partially mediates the relationship between school bullying and depression, anxiety, and stress. In addition, interpersonal relationships and their four dimensions of peer, teacher-student, parent-child, and self relationships moderate the association between core self-evaluation and depression, anxiety, and stress significantly. Among them, the moderating effect on depression is the strongest, and the protective effect of self relationships and parent-child relationships is the most prominent.

In future interventions, educators should focus on addressing anxiety among middle school students and strengthening teacher-student relationships. When addressing school bullying, it is essential not only to stop the bullying behavior itself but also to address the psychological consequences it causes. In addition to stopping the behavior, interventions for school bullying can reduce depression, anxiety, and stress by improving students' core self-evaluation, while compensating for insufficient internal resources by improving interpersonal relationships. Furthermore, personalized mental health interventions should be implemented based on the differing moderating effects of peer, teacher-student, parent-child, and self-relationships on depression, anxiety, and stress. The results of this study not only enrich the research on the correlation between campus bullying and negative emotions among middle school students, but also provide empirical evidence for early prevention and precise psychological intervention.

### 5.1 Limitations and future direction

Firstly, the independent variable in this study was a binary variable (whether one had experienced school bullying), which could only distinguish whether participants had been victimized, and could not subdivide the form, frequency, and severity of bullying, making it difficult to explore the differential effects of different bullying experiences on depression, anxiety, and stress. Future research can employ multidimensional bullying assessment scales to examine in depth the distinct mechanisms by which different types of bullying affect depression, anxiety, and stress of middle school students. Second, this study focused solely on emotional indicators such as depression, anxiety, and stress, and did not include important developmental indicators such as academic performance. Future research can incorporate objective data on academic performance to analyze the association between school bullying and academic performance, and further explore the mechanisms through which academic performance influences the relationship between school bullying and depression, anxiety, and stress. Furthermore, since the current study was cross-sectional in design, it could not establish causal relationships. Future research can adopt a longitudinal design and employ a cross-lagged model to explore the causal relationships and dynamic changes among school bullying, core self-evaluation, interpersonal relationships, and depression, anxiety, and stress. At the same time, intervention studies can be conducted to examine the actual effects of improving core self-evaluation and interpersonal relationships on alleviating depression, anxiety, and stress, providing a basis for mental health interventions on campus. Finally, the survey participants in this study were mainly concentrated in Anhui Province, with a relatively homogeneous regional and educational background. Students from different regions and cultural backgrounds may exhibit varying emotional responses and interpersonal dynamics when faced with school bullying; thus, future research should expand the sampling scope and conduct multi-regional investigations.

## Data Availability

The datasets presented in this article are not readily available because Data is available from the corresponding author upon reasonable request. Requests to access the datasets should be directed to sudan20230906@163.com.
